# More is not always better-association between hip range of motion and symptom severity in patients with femoroacetabular impingement syndrome: A cross-sectional study

**DOI:** 10.1016/j.bjpt.2025.101189

**Published:** 2025-02-19

**Authors:** Diogo A. Gomes, Joshua Heerey, Mark Scholes, Andrea Mosler, Denise Jones, Sally Coburn, Richard Johnston, Rintje Agricola, Michael Girdwood, Marcella Ferraz Pazzinatto, Joanne Kemp

**Affiliations:** a La Trobe Sport and Exercise Medicine Research Centre, La Trobe University, Bundoora 3086, Victoria, Melbourne, Australia; b Barwon Health Research, Geelong 3220, Victoria, Melbourne, Australia; c Orthopaedics, Erasmus MC, Rotterdam, Zuid- Holland, The Netherlands

**Keywords:** Hip, Physical therapy, Rehabilitation

## Abstract

**Background:**

Restricted hip range of motion (ROM) is a common finding in patients with femoroacetabular impingement (FAI) syndrome. However, the association between hip ROM and symptom severity in these individuals is unclear.

**Objective:**

Explore associations between symptom severity and hip flexion and rotation ROM in patients with FAI syndrome and determine if ROM measures can discriminate those with worse symptoms.

**Methods:**

Data from 150 participants with FAI syndrome were analysed. A digital inclinometer was used to measure hip flexion, internal rotation, and external rotation ROM. Symptom severity was quantified using the symptoms subscale of the international Hip Outcome Tool questionnaire (iHOT-Symptoms). Multivariable fractional polynomial analyses explored associations between hip ROM measures and symptom severity. Receiver operator characteristic curves explored the ability of ROM measurements to discriminate participants with different symptomatic states.

**Results:**

Smaller hip flexion ROM values were associated with worse iHOT-Symptoms scores (*p* < 0.01; R^2^=0.242); with the polynomial concave association attenuated at approximately 120° of hip flexion ROM. Hip internal rotation was weakly associated with iHOT-Symptoms score (*p* = 0.01; R^2^=0.033). Hip external rotation ROM was not associated with iHOT-Symptoms score (*p* = 0.06). A hip flexion value of 107° best discriminated mild to moderate and severe symptom states (sensitivity 92%, specificity 52%).

**Conclusion:**

Less hip flexion ROM was associated with worse symptoms in patients with FAI syndrome. Patients with hip flexion ROM ≥ 107° had a 15-fold decrease in the likelihood of having severe symptoms. Hip rotation ROM measures do not have a clinically meaningful association with symptom severity.

## Highlights

• Reduced hip flexion ROM is associated with worse symptoms in patients with FAI syndrome.
• Patients with hip flexion ROM ≥107° had a lower chance of having severe symptoms.
• The increase in the iHOT-symptoms score attenuated at 120° of hip flexion ROM.
• The association between hip internal rotation ROM and symptoms was not relevant.
• Hip external rotation ROM and symptoms were not associated.

## Introduction

Femoroacetabular impingement (FAI) syndrome is a common cause of hip-related pain,^1^ potentially leading to deteriorating physical activity levels, and poor quality of life.^2^, ^3^, ^4^ This condition is characterised by altered femoral (cam morphology) and/or acetabular (pincer morphology) shape which results in premature bony contact during hip movement.^4^ This mechanical abutment may contribute to the reduced hip joint range of motion (ROM) observed in people with FAI syndrome.^5^

Restricted hip ROM is a common treatment target in patients with FAI syndrome.^4^^,^^6^ When compared to asymptomatic individuals, those with FAI syndrome have reduced hip flexion and rotation (internal and external) ROM,^5^ which may contribute to impaired function and symptoms aggravation during common daily activities, such as squatting, sitting, and getting out of a car.^4^^,^^7^ Reduced hip flexion is associated with worse hip symptoms in those undergoing hip arthroscopy for suspected chondrolabral pathology and other causes of hip-related pain, including cam morphology.^8^ However, while reduced hip ROM may relate to self-reported hip function, its association with symptom severity in patients diagnosed with FAI syndrome using recommended criteria guidelines^4^ (presence of symptoms, clinical signs, and imaging findings) seeking non-surgical treatment is unclear.^9^ Knowledge of this association in patients with FAI syndrome can inform future research on the identification of potential rehabilitation targets and development of optimal surgical and non-surgical interventions for these patients.

A spectrum of symptom severity exists in FAI syndrome, with some individuals experiencing milder symptoms while others present more severe manifestations of this condition.^4^ Because it is presumed that hip ROM can influence symptom variation in patients with FAI syndrome, previous studies explored if hip ROM cut-off values were able to discriminate patients with different symptomatic states after arthroscopy to improve rehabilitation.^10^ Findings indicate that patients with ≥ 100° of hip flexion ROM after surgery have a higher probability of presenting a less severe symptomatic state compared to those with flexion ROM values below this cut-off point.^10^ However, there is a paucity of evidence regarding hip ROM cut-off values able to discriminate patients with FAI syndrome with different symptomatic states before treatment. Such knowledge could potentially help clinicians identify hip ROM values that might alleviate symptoms if achieved.

This study aimed to (i) explore the association between symptom severity and hip flexion and rotation (internal and external) ROM in patients with FAI syndrome, and (ii) explore if ROM measures can discriminate patients with FAI syndrome with different symptomatic states.

## Methods

### Study design

This cross-sectional study is reported according to the Strengthening the Reporting of Observational Studies in Epidemiology (STROBE).^11^ This study used baseline data from a randomised controlled clinical trial (RCT) comparing two exercise-based physical therapist-led interventions for FAI syndrome.^12^ In that study, 154 individuals diagnosed with FAI syndrome were randomly assigned to one of two physical therapist-led interventions (targeted strengthening or standardised stretching). Participants with no ROM data were excluded from the present study.

### Participants

Individuals with hip pain from the general community (Victoria, Australia) were screened for eligibility for the primary RCT. Detailed eligibility criteria are reported elsewhere.^12^ Briefly, patients were eligible if they: i) were aged 18 to 50 years; ii) reported hip/groin pain which was aggravated by prolonged sitting or hip movements into positions of impingement; iii) reported hip-related pain ≥3/10 on numerical pain scale for ≥6 weeks; iv) had cam morphology (defined as radiographic alpha angle ≥60°^13^); and iv) had a positive flexion-adduction-internal rotation (FADIR) pain provocation test. Patients were excluded if they: i) had undergone physical therapy for the hip or had an intra-articular hip-joint injection in the preceding 3 months; ii) reported previous hip or back surgery; iii) planned lower limb surgery in the following year; iv) had radiographic hip osteoarthritis (Kellgren and Lawrence score ≥2); v) had a neurological or other musculoskeletal condition; or vi) were unable to understand English. Body mass index (BMI), age, and sex were also recorded. The alpha angle was examined using both anterior-posterior (AP) and Dunn 45° radiographs, and the highest value identified was used for inclusion and data analysis. In case of bilateral hip pain, the most symptomatic side (self-reported) was included.

### Symptom severity

The symptoms subscale from the international Hip Outcome Tool (iHOT-33) questionnaire was used to assess symptom severity. This subscale comprises 16 questions examining the respondent's severity of pain, stiffness, and functional limitations.^14^ Scores for the iHOT-symptoms subscale range from 0 to 100, with higher scores indicating less pain and better function. The iHOT-symptoms subscale presents adequate validity and reliability for the assessment of individuals with hip pain with a standard error of measurement of 7 points.^15^

### Hip range of motion

Methods used for the measurement of active hip ROM are described in detail elsewhere.^12^ Briefly, all tests were measured using a digital inclinometer and participants were instructed to actively move through the relevant motion as far as possible. For all tests, the contralateral leg was restrained using a belt firmly placed over the mid-thigh region to minimise variability in testing position. Hip ROM was measured in degrees and the average value of three repetitions was used for analysis. Hip internal and external rotation ROM were assessed with the participant sitting on the end of a plinth (90° of hip flexion). Hip flexion ROM was assessed with the participant in a supine position. The specific hip ROM tests were selected due to their excellent intra and inter-rater reliability (intraclass correlation coefficients ranging from 0.76 to 0.97) and ease of use in clinical practice.^16^^,^^17^

### Statistical analysis

Data analyses were conducted using R software (R Core Team, 2016). Shapiro-Wilk tests and visual inspections of histograms were performed to assess data normality. Continuous demographic data were summarised using mean and standard deviation or median and interquartile range, as appropriate. Independent *t*-tests were performed to investigate between-group differences in age, body mass index (BMI), and alpha for the two groups established to dichotomise participants’ symptomatic state. A chi-squared test was performed to test the difference in sex prevalence between groups.

#### Primary aim: association between hip ROM and iHOT-Symptoms

Separate linear regression models were built to explore associations between each combination of iHOT-symptoms scores (dependent variable – scores between 0 and 100) and the independent variables of hip flexion ROM, external rotation ROM, and internal rotation ROM. Models were assessed for violations of assumptions. Residual scatter plots were used for the assessment of linearity and homoscedasticity. Graphical assessments and Shapiro-Wilk tests were used to assess normality of residuals.

Non-linear associations between dependent and independent variables were explored using multivariable fractional polynomial (MFP) analyses, and the model functional form (linear or non-linear i.e., quadratic, cubic, square root, etc.) that best fitted the relationship between variables was selected.^18^ The MFP closed test method was used to compare residual deviance between models with different functional forms and for model selection.^18^^,^^19^ For each combination of variables, four model forms were generated and compared; i) null model, ii) linear model, iii) best fitting first-degree fractional polynomial model, and iv) best fitting second-degree polynomial model. Detailed information on model selection using the closed test method is available in the Supplementary material A available online.

Selected models were analysed unadjusted and adjusted for the covariates of age, BMI, sex, and alpha angle. Interaction effects between participants’ hip ROM measurements and sex (sex*ROM) and alpha angle (alpha angle*ROM) were explored and removed from the models if not significant. If there was no significant difference in model fit between unadjusted and adjusted models, unadjusted models were presented.^15^ Influential outliers were identified through residual scatter plots and Cook's distance. If modelled relationships were unduly influenced by individual points, exclusion criteria values were created using median absolute deviation (median – (2*median absolute deviation)).^20^ If individual values from outliers exceeded the developed criteria, outliers were excluded from the main analysis. Pseudo R^2^ values were used to quantify the strength of the modelled relationships. Level of significance was set at 0.05.

#### Secondary aim: ability of ROM measurements to discriminate different symptomatic states

If associations between iHOT-symptoms scores and hip ROM measurements existed, receiver operator characteristic (ROC) curves were used to explore the ability of ROM measurements to discriminate participants with different symptomatic states. Participants were stratified into two groups according to their iHOT-symptoms scores: severe hip symptoms (iHOT-symptoms score < 63 points) and mild to moderate hip symptoms (iHOT-symptoms score ≥ 63 points).^8^ The criteria used for group stratification was developed in a previous study through partitioning around medoids cluster analysis. The area under the ROC curve (AUC) was used to assess and classify discriminative ability as: no ability (AUC=0.5); poor (0.5 < AUC < 0.7); acceptable (0.7 < AUC < 0.8); excellent (0.8 < AUC < 0.9); and outstanding (AUC ≥ 0.9).^21^ In case of at least acceptable discriminative ability, the best ROM cut-off value for groups discrimination was identified using the Youden index (J= sensitivity + specificity – 1), with a higher index score indicating better combined sensitivity and specificity.^22^ Positive (LR+:Sensitivity/1 – specificity) and negative (LR-:1 – sensitivity/specificity) likelihood ratios and post-test probability (Pre-test odds x likelihood ratio) were calculated as appropriate.^23^

## Results

Of the 154 participants in the original clinical trial, 150 participants with FAI syndrome were included in the present study. Characteristics of excluded participants are presented in the Supplementary material B available online**.** Four participants were excluded due to missing ROM data (*physical assessment not completed due to COVID-19 lockdown*). One participant had missing data for hip flexion ROM, and 10 participants had missing data for hip external and internal rotation ROM. Therefore, analyses involving hip flexion ROM and hip internal and external rotation ROM included 149 and 140 participants, respectively. Seventy-four participants (49%) had severe symptoms and 76 participants had mild to moderate symptoms. Table 1 summarises participant characteristics. Age (*p* = 0.52), alpha angle (*p* = 0.33), and the proportion of male and female participants (*p* = 0.07) did not differ between the mild to moderate and severe symptom groups. Participants with severe symptoms had greater BMI compared to participants with mild to moderate symptoms (*p* < 0.05).

**Table 1:** Characteristics of participants with FAI syndrome with different symptomatic conditions.

Participants characteristics	FAI syndrome (*n* = 150)	Mild to moderate symptoms (*n* = 76)	Severe symptoms (*n* = 74)
Age (years)	35 (9)	34 (10)	35 (9)
Sex (female)	76 [52%]	36 [47%]	40 [54%]
BMI (kg/m^2^)*	25.5 (5.0)	24.6 (4.1)	26.5 (5.6)
Alpha angle (^o^)	73 (7)	72 (7)	73 (8)
iHOT-symptoms	61 (17)	75 (7)	47 (11)
Hip flexion ROM (^o^)^a^	113 (13)	119 (10)	107 (13)
Hip ER ROM (^o^)^b^	33 (7)	34 (7)	32 (8)
Hip IR ROM (^o^)^b^	28 (8)	30 (8)	27 (8)

Values are presented as mean (standard deviation). For sex, values are presented as number of participants [proportion]. Sample size variations: *a* = 149, *b* = 140. * indicates significant difference between symptomatic conditions group. BMI, body mass index; ER, external rotation; iHOT, international Hip Outcome Tool; IR, internal rotation; ROM, range of motion.

### Model forms and final model selection

Interaction terms were not included in the models because no significant sex*ROM and alpha angle*ROM interactions existed in any tested models (*p* > 0.05). Interaction plots are presented in the Supplementary material C available online**.** Influential outlying data points were found for one model only. For the association between iHOT-Symptom scores and hip external rotation ROM, three influential outlying data points were excluded. Outlying values, participant characteristics, criteria for exclusion, and the model that includes the outlying data points are presented in the Supplementary material D available online.

Comparison of model forms and final model selection are summarised in Table 2. There was no evidence of an association between iHOT-symptoms score and hip external rotation ROM (no significant difference was found between best fitting fractional second-degree polynomial model and null model [p-null = 0.062]). For the association between iHOT-symptoms scores and hip flexion ROM, a first-degree quadratic polynomial regression model was best-fitting (p-fp = 0.355). For the relationship between iHOT-symptoms scores and hip internal rotation ROM, a linear regression model was indicated (p-lin = 0.167). Significance levels did not differ between unadjusted and adjusted models (Supplementary material E available online). Therefore, unadjusted models were selected for data interpretation.

**Table 2:** Comparisons of model forms and model selection.

Independent variable	Model class (power)	Residual deviance	p-value
Hip flexion ROM	Null (0)	43,537.92	p-null < 0.00
	Linear (1)	34,523.68	p-lin = 0.03
	**Quadratic polynomial (−2)**	**33,029.04**	**p-fp = 0.35**
Hip internal rotation ROM	Null (0)	42,132.43	p-null = 0.02
	**Linear (1)**	**40,428.85**	**p-lin = 0.16**
	Cubic polynomial (3)	38,994.52	p-fp = 0.92
Hip external rotation ROM	**Null (0)**	**38,785.49**	**p-null = 0.06**
	Linear (1)	38,498.46	p-lin = 0.04
	Quadratic polynomial (−2)	37,574.49	p-fp = 0.09

For all models the dependent variable was the iHOT-symptoms score. p-null refers to comparisons between null models and best-fitting fractional polynomial models. p-lin refers to comparisons between linear models and best-fitting fractional polynomial models. p-fp refers to comparisons between first and second-degree transformation fractional polynomial models. Bold values: models selected for data analysis.

### Associations between iHOT-symptoms scores and hip ROM values

**Fig. 1:**
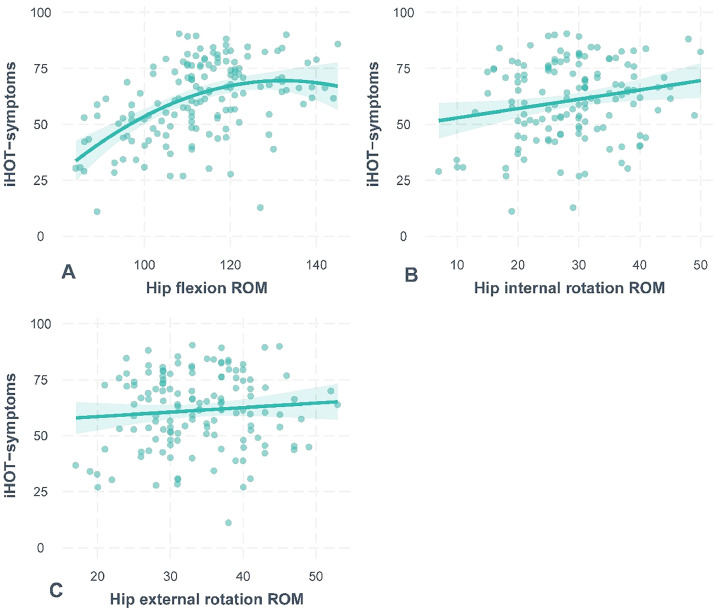
(A) Association between iHOT-symptoms score and hip flexion range of motion (*n* = 149). (B) Association between iHOT-symptoms score and hip internal rotation range of motion (*n* = 140). (C) Association between iHOT-symptoms score and external rotation range of motion. Associations were not modified by alpha angle and sex. ROM=range of motion.

A polynomial (concave) association was found between the iHOT-symptoms score and hip flexion ROM (estimate: −41.35; 95% CI: −53.20, −29.50; *p* < 0.001; R2=0.242), where smaller hip flexion ROM values were associated with lower (worse) iHOT-symptoms scores, and the rate of increase in iHOT-symptoms scores attenuated at greater values of hip flexion ROM (Fig. 1a). Calculating equation is presented in the Supplementary material F available online. A positive linear association was found between iHOT-symptoms scores and hip internal rotation ROM (estimate: 0.41; 95% CI: 0.07, 0.75; *p* = 0.017; R2: 0.033) (Fig. 1b), where a 1° increase in hip internal rotation ROM was associated with a 0.41-point increase in iHOT-symptoms scores. There was no association between iHOT-symptoms score and hip external rotation ROM (p-null = 0.062).

### Hip ROM discriminative ability – ROC curve analyses

**Fig. 2:**
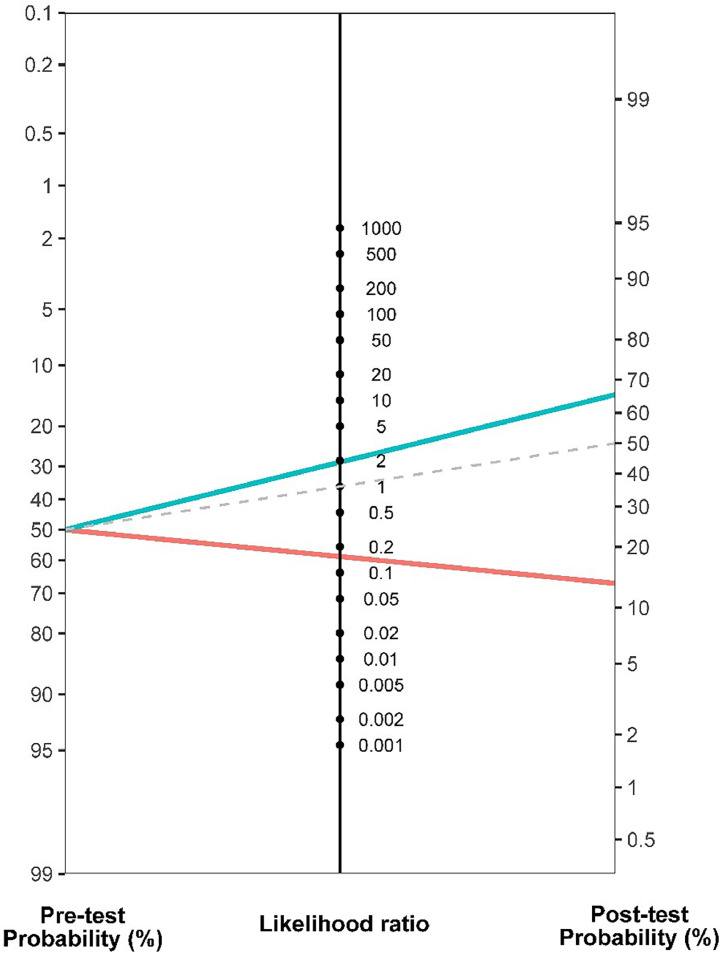
Nomogram demonstrating the pre-test and post-test probability of having severe symptoms based on a hip flexion ROM test. Gray line (dashed) represents no change from pre-test to post-test probability (likelihood ratio = 1). The probability of having severe symptoms decreases from 49% to 13% following a hip flexion ROM test ≥ 107° (red line). The probability of having severe symptoms increases from 49% to 65% following a hip flexion ROM test ≤ 107° (blue line).

Hip flexion ROM demonstrated acceptable ability to discriminate participants with different symptomatic states (AUC: 0.77; standard error: 0.03; 95% CI: 0.69, 0.84; *p* < 0.001). The best hip flexion ROM cut-off score for discrimination was 107° (J: 0.44), with a sensitivity of 92% (95% CI: 83%, 96%) and a specificity of 52% (95% CI: 41%, 63%). Participants with ≥ 107° of hip flexion ROM had a 15-fold decrease in the likelihood of having severe symptoms (LR-: 0.15). The post-test probability of 0.13 indicates that when hip flexion ROM was ≥ 107°, the probability of having severe symptoms dropped by 36% (Fig. 2).

ROC analysis indicated no ability of hip internal rotation ROM to discriminate participants with FAI syndrome with different symptomatic states (AUC: 0.56; standard error: 0.04; 95% CI: 0.46, 0.65; *p* = 0.186).

## Discussion

Our study of patients with FAI syndrome found that greater hip flexion ROM values were associated with less severe symptoms. However, the increase in the iHOT-symptoms score attenuated at approximately 120° of hip flexion ROM. We found a small association between hip internal rotation ROM and symptoms, however, this only explained 3% of the variance in symptoms and had questionable clinical utility. Hip external rotation ROM was not associated with symptoms.

Reduced hip flexion ROM seems to be an indicator of worse symptom severity in those with FAI syndrome, explaining 24% of symptom variance. Similar findings have been observed in patients pre-and post-hip arthroscopy,^8^^,^^10^ suggesting that the relationship is maintained across the clinical spectrum of FAI syndrome. Hip flexion ROM restriction may have a significant impact in activities of daily living (ADLs) for people with FAI syndrome. Many ADLs require large ranges of hip flexion ROM, such as maintaining a seated position, getting out of a car, and putting socks and shoes on, which may exacerbate symptoms in patients with FAI syndrome.^4^ These tasks require hip flexion joint angles from 95 to 121°^7^^,^^24^ and as the mean (SD) peak hip flexion ROM for those with severe symptoms was 107° (SD 13), it is likely that these tasks require almost all or more of the patients’ available flexion ROM. Notably, the association between iHOT-symptoms score and hip flexion ROM attenuated at approximately 120° of hip flexion ROM (Fig. 1). This may be explained by the fact that the amount of hip flexion ROM value may be enough for patients to perform these functional tasks comfortably. Performing functional tasks with greater ease and comfort are common goals of patients with FAI syndrome. Interventions focused on increasing hip flexion ROM may also improve hip symptoms in those with limited ROM, helping patients achieve their goals and potentially increasing treatment satisfaction.

Our findings can assist clinicians to select patients who may benefit from interventions that aim to improve hip ROM. Results from the ROC analysis indicate that patients with FAI syndrome and ≥ 107° of hip flexion ROM had a reduced likelihood (15-fold) of having severe symptoms, indicating that those with lower ROM values (<107°) may benefit from improvements in hip flexion ROM. However, more is not always better, with the rate of increase in the iHOT-symptoms score diminishing at approximately 120° of flexion ROM. This suggests that patients with >120° of hip flexion ROM might not benefit from interventions focused on improving hip flexion ROM. Improving hip flexion ROM in patients with FAI syndrome to between 107 and 120° may be clinically feasible during rehabilitation.^12^^,^^25^ Hip arthroscopy and a physical therapist-led intervention can, on average, improve hip flexion ROM by 9 and 14°, respectively, in patients with FAI syndrome.^12^^,^^25^ To confirm importance of our findings, sufficiently powered randomised controlled trials with adequate methodological quality should explore if the effect of physical therapist-led interventions on hip-related symptoms, is mediated by improvements in hip flexion ROM.

Reduced hip rotation ROM might not be a relevant target of interventions for patients with FAI syndrome. Consistent with previous studies,^8^^,^^10^ hip internal rotation ROM explained only 3% of the variance in symptoms in patients with FAI syndrome, while hip external rotation ROM was not associated with symptoms. Based on our results, an increase of 19° of hip internal rotation ROM is needed to achieve a minimal important change in the iHOT-symptoms score (8 points).^15^ Both surgical and conservative interventions might be ineffective at altering hip rotational ROM in patients with FAI syndrome.^25^, ^26^, ^27^ This may be partially explained by the presence of other hip morphological features (e.g., acetabular and femoral malversion) that can restrict rotational ROM.^28^^,^^29^ Therefore, clinicians need to consider the merits of targeting hip rotational ROM impairments with hip arthroscopy and exercise-based treatments as they may not have clinically meaningful effects for patients with FAI syndrome.

Our findings indicate that cam morphology size did not alter the relationships between hip ROM and symptom severity in patients with FAI syndrome. Some studies report an association between larger cam morphology and reduced ROM^28^^,^^30^ and more severe symptoms,^31^, ^32^, ^33^ while other studies report no association between these variables.^34^, ^35^, ^36^, ^37^ However, none of these studies (including the present) considered other bony hip morphology characteristics that can be concomitantly present with cam morphology and possibly influence hip ROM and symptoms of patients with FAI syndrome, which may explain the conflicting results. Femoral and acetabular retroversion, and protusio acetabuli are associated with reduced hip ROM and worse functional/symptomatic state.^29^^,^^30^^,^^38^, ^39^, ^40^ Also, the lack of influence of cam morphology size on the associations between hip ROM and symptoms severity in the present study could be explained in part by the use of active hip ROM testing methods that did not control for factors such as spinopelvic movement. However, the decision to assess active rather than passive hip ROM in this study was based on the hypothesis that active hip ROM might better reflect the impaired daily activities of patients with FAI syndrome. Future studies should explore the influence of different bony hip morphologies on hip ROM (active and passive) and symptoms of patients with FAI syndrome.

Our study has limitations. Given the cross-sectional nature of the study, it is not possible to indicate a causal relationship between hip flexion ROM and symptom severity, and results should be interpreted with caution. Our results regarding the influence of cam morphology size on the association between hip ROM and symptom severity can be influenced by the method used to assess cam morphology, as the highest alpha angle value from either the anteroposterior pelvis or Dunn 45° radiograph was considered for data analysis. The association between hip ROM and symptoms may be influenced by the presence of other bony hip morphologies not assessed in this study, such as the pincer morphology and the acetabular version. Also, to undertake the ROC curve analysis, the iHOT-symptoms score was used to dichotomize participants into two groups with different symptomatic states. However, the iHOT-symptoms score ranges from 0 to 100 points and was not originally developed to be used as a dichotomous variable. This prevented us from investigating participants with symptom severity levels different than the ones explored in our study.^4^

## Conclusion

Reduced hip flexion ROM was associated with worse symptoms and explained 24% of the variance of symptoms of patients with FAI syndrome. Patients with >107° of hip flexion ROM had a 15-fold decrease in the probability of having severe symptoms on the iHOT-Symptoms Subscale. The association between hip internal rotation ROM and symptoms was not clinically relevant, while hip external rotation ROM and symptoms were not associated.

## Declaration of competing interest

The authors have no competing interest to declare.

## Appendix Supplementary materials

Image, application 1
