# Supplementary material for: More is not always better-association between hip range of motion and symptom severity in patients with femoroacetabular impingement syndrome: A cross-sectional study
Source: Braz J Phys Ther. 2025 Feb 19;29(2):101189. doi: 10.1016/j.bjpt.2025.101189 (PMC11883298; doi:10.1016/j.bjpt.2025.101189)
Supplement: Supplementary file 1 [file mmc1.pdf]

Supplementary online materials

Online material A

Model selection using closed test method from multivariable fractional polynomial analysis

Non-linear associations between dependent and independent variables were explored using multivariable fractional polynomial (MFP) analyses and the model functional form (linear or non-linear i.e., quadratic, cubic, square root, etc.) that best fitted the relationship between variables was selected. The MFP "closed test" method was used to compare residual deviance between models with different functional forms and for model selection. For each combination of variables, four model forms were generated and compared; i) null model, ii) linear model, iii) best fitting fractional first-degree polynomial model, and iv) best fitting fractional second-degree polynomial model. The closed test method consists of three steps. Step one – inclusion test: Residual deviance was compared between the best-fitting second-degree fractional polynomial model and the null model, where no difference (p-null > 0.05) indicated no association between variables. If a statistically significant difference existed (p-null < 0.05) step two was conducted. Step two – non-linearity test: residual deviance between the multifractional polynomial and linear model was compared. If the residual variance did not differ (p-lin > 0.05) between model forms, the linear model was considered the best fit to describe the relationship between variables. When residual deviance differed between the two model forms (p-lin < 0.05), step three was conducted. Step three – simplification test: first and second-degree best-fitted fractional polynomial models were compared. Second-degree models were selected for data analysis when a difference existed (p-fp < 0.05); otherwise, first-degree transformation models were selected.

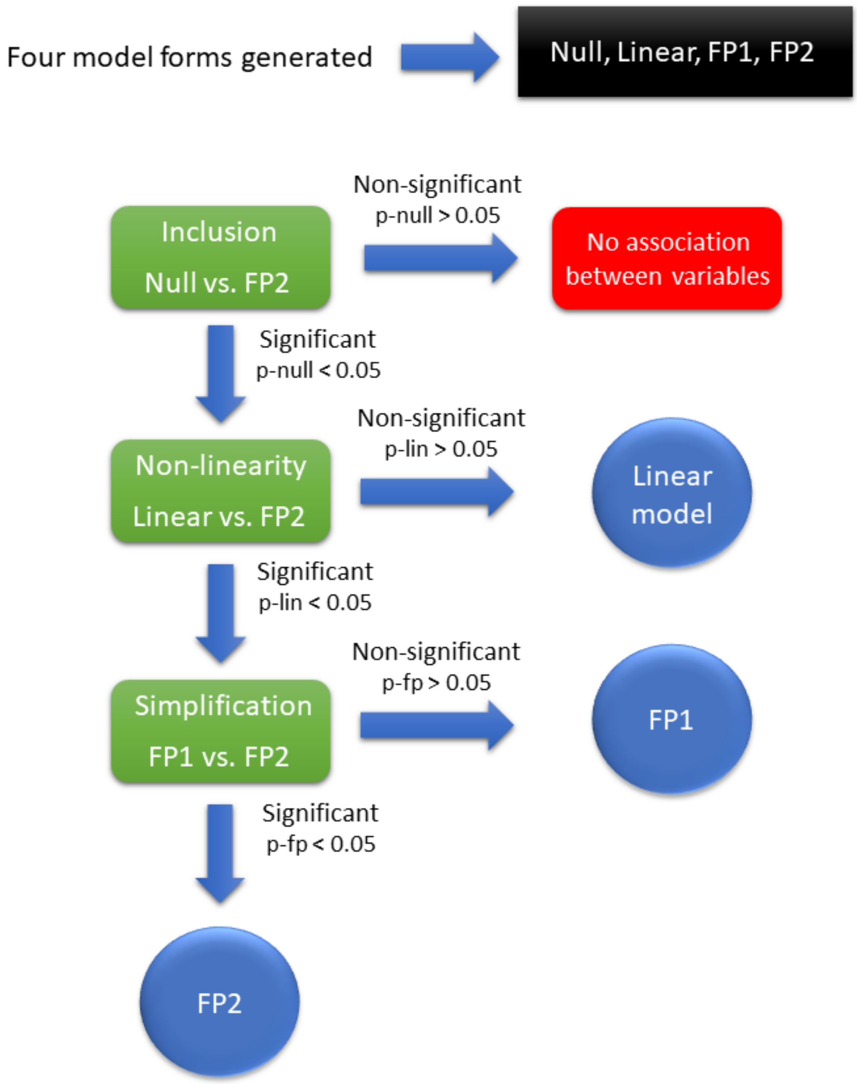

Figure 1. Multivariable fractional polynomial analysis closed test method for model selection. FP1; best fitting first-degree polynomial model. FP2; best fitting second-degree polynomial model. p-null refers to comparisons between FP2 and null models. p-lin refers to comparisons between FP2 and linear models. p-fp refers to comparisons between FP1 and FP2 models. Figure adapted from Zhang (2016).

Online material B

Characteristics of participants with missing data

	Included participants (n = 150)	Hip flexion ROM missing data (n = 1)	Hip external rotation ROM missing data (n = 10)
Age (years)	35 (9)	40 (NA)	32 (6)
Sex (female)	76 [52%]	1 [100%]	7 [70%]
BMI (kg/m^2)	25.5 (5.0)	21.0 (NA)	23.7 (3.2)
Alpha angle (°)	73 (7)	66 (NA)	71 (7)
iHOT-symptoms	61 (17)	64 (NA)	67 (10)

Values are presented as mean (standard deviation). BMI, body mass index; iHOT, internation Hip Outcome Tool; ROM, range of motion. For sex, values are presented as number of participants [proportion]. One participant had missing data for hip flexion ROM and 10 participants for hip external and internal rotation ROM.

Online material C

Interaction plots

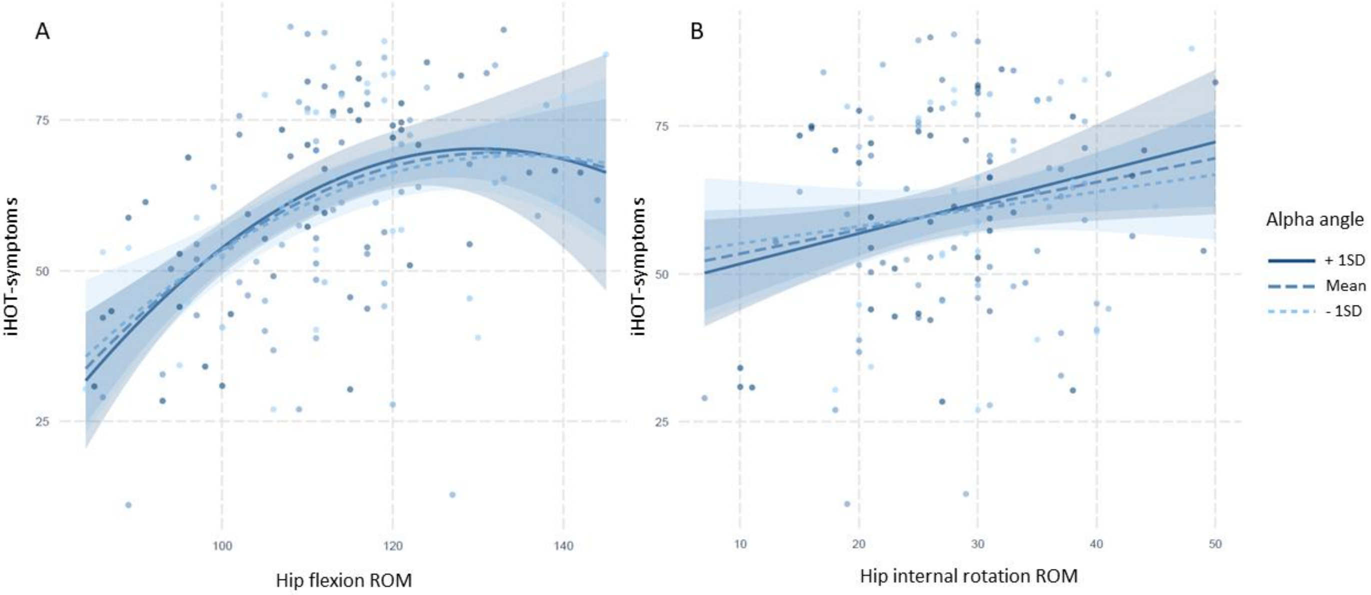

Figure 2. Regression models exploring the association between iHOT-symptoms score and hip flexion ROM (A), and (B) hip internal rotation ROM using alpha angle as interaction factor. There was no interaction between alpha angle and hip flexion ROM (p = 0.623) and between alpha angle and hip internal rotation ROM (p = 0.474) in both models.

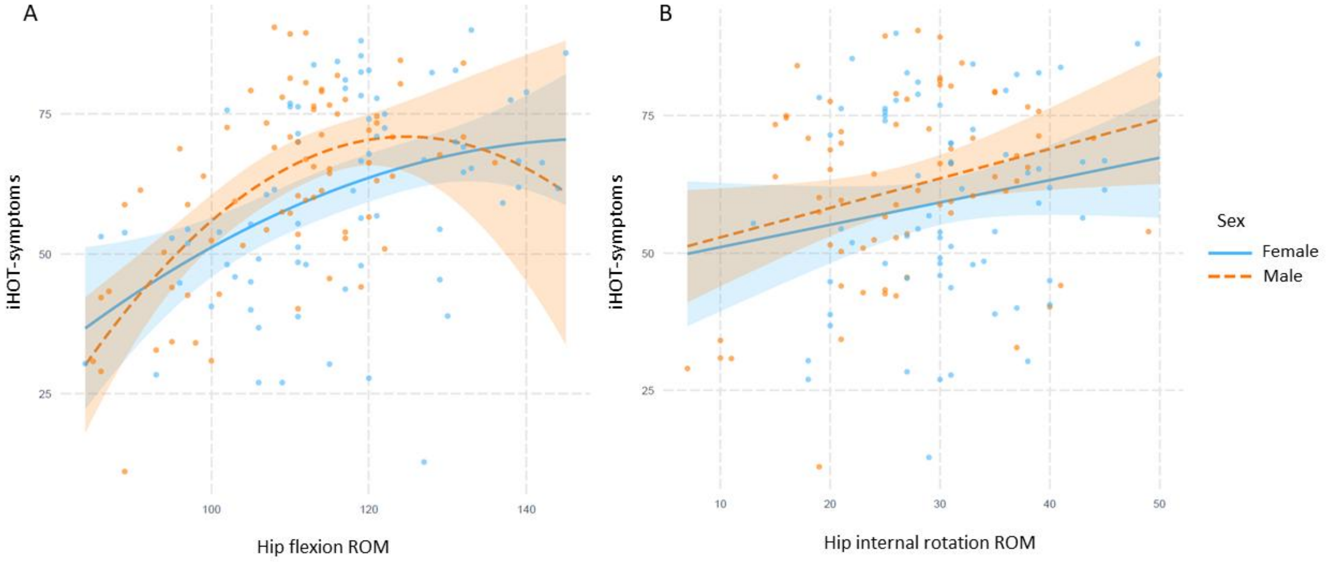

Figure 3. Regression models exploring the association between iHOT-symptoms score and hip flexion ROM (A), and (B) hip internal rotation ROM using sex as interaction factor. There was no interaction between sex and hip flexion ROM (p = 0.178) and between sex and hip internal rotation ROM (p = 0.717) in both models.

Online material D

Table 3. Regression models exploring the association between iHOT-symptoms scores (dependent variable) and hip external rotation range of motion (independent variable) with and without identified outliers

	Funcional form	Regression coefficient (SE)	Confidence interval (95%)	t-value	p-value	Adjusted R^2
Model with outliers	Quadratic polynomial	-0.046 (0.013)	-23.15 to -6.19	-3.337	**p < 0.001**	0.0941
Model without outliers	Linear	0.201 (0.199)	-0.19 to 0.59	1.007	p = 0.316	0.0001

For the analysis without outliers three individual points were excluded. SE; standard error. Bold values highlight significant associations between variables.

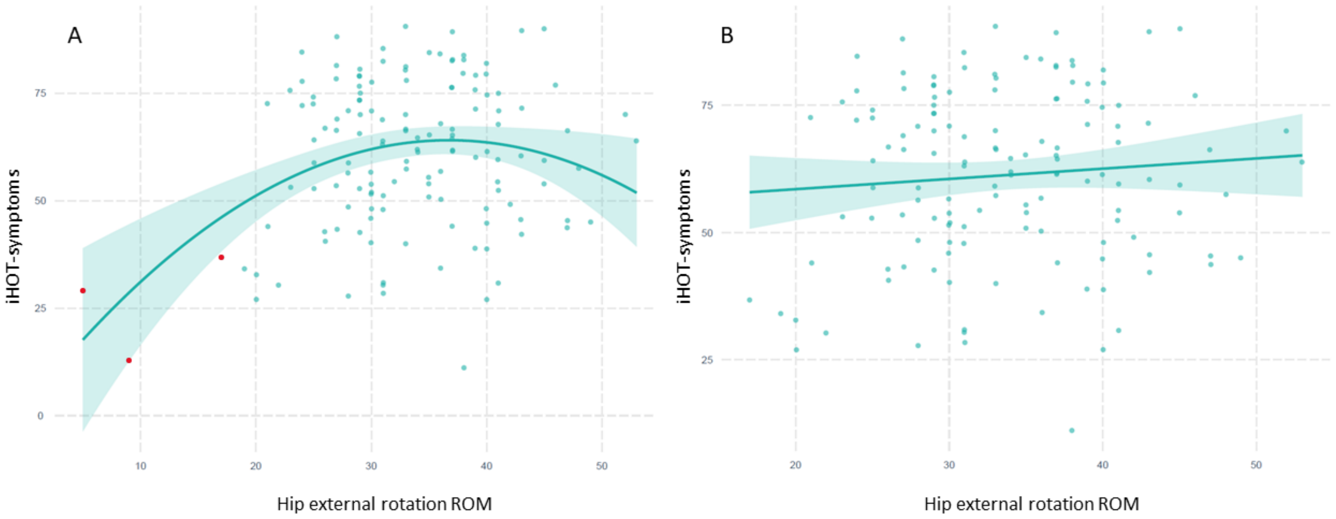

Figure 4. Regression models exploring the association between iHOT-symptoms scores and hip external rotation range of motion with (A) and without (B) identified outliers. ROM, range of motion. Red dots highlight identified outliers.

Characteristics of individual influential outliers removed from main analysis

	Outlier #1	Outlier #2	Outlier #3
Age (years)	39	39	45
Sex	Male	Female	Female
BMI (kg/m^2)	34	25	24
Alpha angle (°)	73	70	72
iHOT-symptoms	29	13	37
External rotation ROM (°)	5	9	17

BMI, body mass index; iHOT, international Hip Outcome Tool; ROM, range of motion.

Absolute median deviation method used to exclude influential outliers:

33 – (2*7) = 19

33: Median; 7: Median absolute deviation; 2: Rejection criterion value.

Influential values lower than 19° of hip external rotation range of motion were excluded from the main analysis.

Online material E

Models exploring relationships between participants iHOT-symptoms scores (dependent variable) and hip range of motion (independent variable)

	Hip flexion ROM	Hip internal rotation ROM
Unadjusted model	**R^2 = 0.242**<br>**p < 0.001**	**R^2 = 0.033**<br>**p = 0.045**
Adjusted model	**R^2 = 0.269**<br>**p < 0.001**	**R^2 = 0.081**<br>**p = 0.017**

Adjusted models were adjusted for the covariates of sex, age, alpha angle, and BMI. Bold values highlight significant associations between variables. ROM, range of motion

Adjusted models exploring relationships between participants iHOT-symptoms scores (dependent variable) and hip range of motion (independent variable)

Independent variable	Term	Estimate	conf.low	conf.high	SE	p-value	t-value
Hip internal rotation ROM	Hip internal rotation ROM	0.366	0.007	0.724	0.181	0.045	2.01
	Age	-0.099	-0.392	0.193	0.148	0.503	-0.67
	BMI	-0.793	-1.347	-0.239	0.280	0.005	-2.83
	Alpha angle	-0.095	-0.476	0.284	0.192	0.618	-0.49
	Sex (male)	4.191	-2.017	10.401	3.139	0.184	1.33
Hip flexion ROM	Hip flexion ROM	-0.013	-0.023	-0.003	0.005	0.011	-2.56
	Age	-0.272	-0.529	-0.015	0.130	0.038	-2.09
	BMI	-0.093	-0.627	0.439	0.269	0.728	-0.34
	Alpha angle	-0.029	-0.352	0.293	0.163	0.858	-0.17
	Sex (male)	4.394	-0.939	9.727	2.698	0.105	1.62

conf.low and conf.high represent estimates 95% confidence intervals. BMI, body mass index; ROM, range of motion

Online material F

Polynomial equation for the association between iHOT-symptoms score and hip flexion ROM

iHOT-symptoms score = 95*hip flexion ROM + 95*(hip flexion ROM)^2 – 41

Term	Estimate	Standard error	t-value	p-value	95%CI
poly (hip flexion ROM,2)1	94.96	5.09	18.65	$p < 0.01$	84.98, 104.93
poly (hip flexion ROM,2)2	-41.35	6.04	-6.83	$p < 0.01$	-52.20, -29.50

ROM, range of motion. 95% CI, estimate confidence interval
